# Integrated Myocardial and Plasma Lipidome Across the Human Obesity–Heart Failure Spectrum

**DOI:** 10.21203/rs.3.rs-10157404/v1

**Published:** 2026-07-17

**Authors:** David J. Polhemus, Annie Butt, Craig Resch, Kathryn Tsai, Vatsa A. Batra, Shuyi Yan, Atharva Mulay, Cinthia Drachenberg, Flora Sam, Traci T Goodchild, David J. Lefer, Mariam Meddeb, Kenneth B. Margulies, Kenneth C. Bedi, Kavita Sharma, Navid Koleini, Amir Ravandi, David A. Kass

**Affiliations:** 1 Division of Cardiology, Department of Medicine, Johns Hopkins University School of Medicine, Baltimore, MD, USA.; 2 Department of Biomedical Engineering, Johns Hopkins University, Baltimore, MD, USA.; 3 Cardiovascular Lipidomics Laboratory, St. Boniface Hospital, Albrechtsen Research Centre, Manitoba, Canada.; 4 Department of Pathology, University of Maryland, Baltimore, MD, USA.; 5 Whitaker Cardiovascular Institute, Boston University School of Medicine, Boston, MA.; 6 Department of Cardiac Surgery, Smidt Heart Institute, Cedars-Sinai Medical Center, Los Angeles, CA.; 7 Cardiovascular Institute, Department of Medicine, Perelman School of Medicine at the University of Pennsylvania, Philadelphia, PA, USA.

**Keywords:** HFpEF, HFrEF, myocardial lipidomics, ceramides, cardiolipin, acylcarnitines, lipotoxicity, metabolic remodeling

## Abstract

Heart failure with preserved ejection fraction (HFpEF) has become the dominant heart failure phenotype, with many patients impacted by marked obesity. Lipid dyshomeostasis is thought to contribute, but human myocardial lipidomic data remain lacking. We performed untargeted lipidomics (>1,000 species) of ventricular samples for non-failing (NF) controls (n=40), HFpEF (n=29), and HFrEF (n=13) patients, integrating results with myocardial transcriptomics, ultrastructure, and plasma lipidomics, across a broad obesity spectrum. Multiple lipid classes increased in HFpEF myocardium including phospholipids, sphingolipids, neutral lipids, and free fatty acids, but acylcarnitines declined. HFrEF had far fewer lipid changes, and cardiolipin and phosphatidylethanolamine depletion. Lipids were minimally altered in NF hearts from obese individuals. Plasma lipidomics identified few inter-group disparities. Heart lipidomics from major animal HFpEF models (high fat diet+L-NAME, ZSF-1 rat, obese-DOCA-salt pig) all differed from human HFpEF. Thus, human HFpEF myocardium has distinctive cardiac lipid accumulation not found with obesity alone or HFrEF, nor mirrored by common preclinical HFpEF models.

## INTRODUCTION

Heart failure with preserved ejection fraction (HFpEF) accounts for more than half of all incident heart failure cases worldwide, with a five-year mortality exceeding 50% and few effective disease-modifying therapies^1,2^. Over the past two decades, the syndrome has undergone a major transition from emphasizing hypertension and left ventricular hypertrophy to one characterized by obesity, metabolic syndrome, systemic inflammation, and multiorgan dysfunction^3,4^. This phenotypic evolution has intensified interest in understanding the metabolic underpinnings of HFpEF, yet major features, notably the lipidome of human hearts, remain largely unknown.

Lipids play multiple roles in cardiac physiology and disease. They constitute the structural scaffold of mitochondrial and sarcolemmal membranes, regulate electron transport chain efficiency through cardiolipins, transduce intracellular signals via ceramides and diacylglycerols, and serve as the dominant oxidative fuel of healthy myocardium^5,6^. Dysregulation of lipid homeostasis, whether through excess accumulation, impaired oxidation, or aberrant membrane remodeling, can precipitate mitochondrial dysfunction, insulin resistance, apoptosis, and contractile failure^7,8^. A prevailing lipotoxicity hypothesis posits that obesity drives myocardial lipid overload, which in turn triggers HFpEF through a cascade of ceramide-mediated cellular injury, mitochondrial uncoupling, and myocardial steatosis^9,10^. While appealing, this model relies largely on animal data and has not yet been rigorously examined in human myocardium across the obesity spectrum in the presence or absence of heart failure. Prior human myocardial studies used few controls, lacked broad lipid class coverage, and did not include comparisons to patients with heart failure with reduced ejection fraction (HFrEF)^11–15^. The extent to which severe obesity without HF induces analogous lipid abnormalities or whether pre-clinical HFpEF models capture changes found in the human disease is also unknown.

Here, we report the first comprehensive myocardial lipidomic characterization of human HFpEF, HFrEF, and non-failing hearts spanning the lean-to-severely-obese BMI spectrum. Using ventricular samples from 82 phenotyped individuals, we quantified over 1,000 lipid species across 21 lipid classes and compared these data with clinical hemodynamics and plasma lipidomics, and myocardial lipidomics from animal models of obesity and heart failure.

## RESULTS

### Myocardial Lipidomics Establishes Phenotype-Specific Lipid Remodeling

Clinical demographics and features of the two HF cohorts and non-failing controls are provided in Extended Table 1. HFpEF patients had higher median BMI compared to NF (37.3 vs 29.9 kg/m^2^, p=0.019), a greater prevalence of hypertension (86% vs. 45%, p<0.001), and higher rates of lipid-lowering therapy (79% vs. 15%, p<0.001), while HFrEF patients had reduced LVEF (15% vs 65%, p<0.001) and higher pulmonary artery systolic pressures (47 vs. 33.5 mmHg, p=0.017) compared to HFpEF. The non-failing cohort spanned a broad BMI range (13.7–51.4 kg/m^2^), enabling interrogation of obesity effects independent of heart failure.

**Fig. 1.**
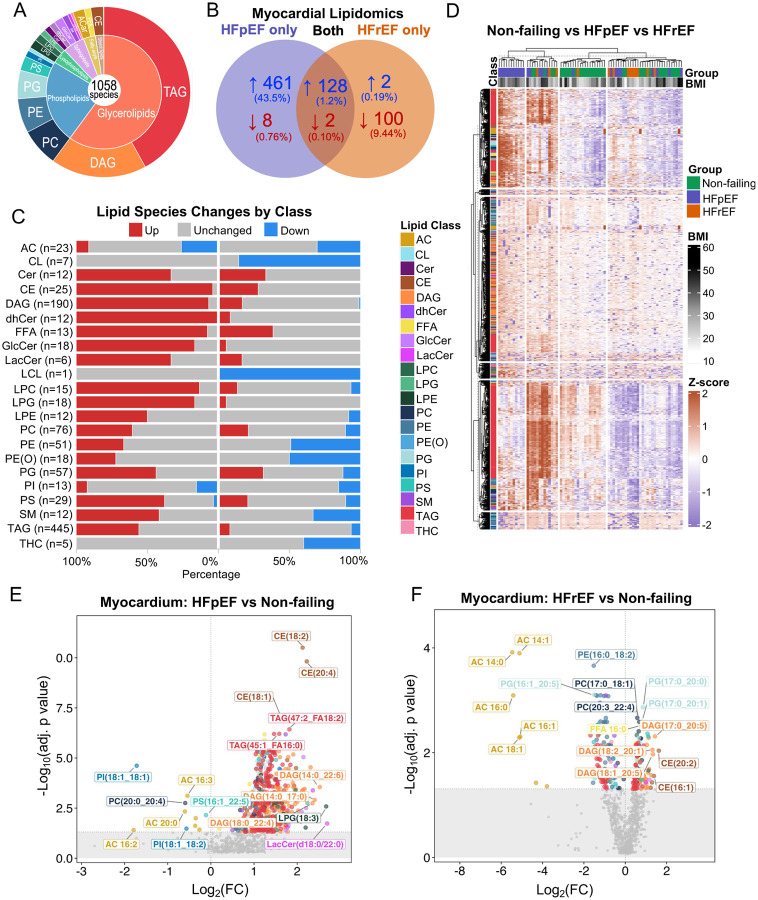
Comprehensive myocardial lipidomics separates HFpEF, HFrEF, and non-failing hearts. (a) Overview of 1,058 lipid species measured across 21 major lipid classes from human ventricular tissue. Fraction of pie chart is proportional to the total number of individual species measured within each class. (b) Venn diagram depicting the number of significantly altered lipid species (Benjamini–Hochberg FDR <5%) uniquely changed in HFpEF vs. NF, uniquely changed in HFrEF vs. NF, and shared between both comparisons. (c) Bar plot showing the percent of significantly downregulated (blue), upregulated (red), and unchanged (grey) lipid species within each lipid class for HFpEF vs. NF and HFrEF vs. NF. (d) Unsupervised hierarchical clustering heatmap of log_2_-transformed, z-score-normalized lipid abundances. Sample annotations: NF (green), HFpEF (purple), HFrEF (red). BMI severity depicted in greyscale. Lipid class is color coded on the left of the heatmap. (e) Volcano plot of HFpEF vs. NF myocardial lipidomics. (f) Volcano plot of HFrEF vs. NF myocardial lipidomics. Horizontal dashed line: FDR=5%; vertical dashed lines: log_2_ FC=0. Lipid species abbreviations: acylcarnitines (AC), cardiolipins (CL), ceramides (Cer), cholesterol esters (CE), diacylglycerols (DAG), dihydroceramides (dhCer), free fatty acids (FFA), glucosylceramides (GlcCer), lactosylceramides (LacCer), lysophosphatidylcholines (LPC), lysophosphatidylglycerols (LPG), lysophosphatidylethanolamines (LPE), phosphatidylcholines (PC), phosphatidylethanolamines (PE), Alkylphosphatidylethanolamine (PE(O)), phosphatidylglycerols (PG), phosphatidylinositols (PI), phosphatidylserines (PS), sphingomyelins (SM), triacylglycerols (TAG), trihexosylceramides (THC)

Untargeted lipidomics identified 1,058 lipid species spanning 21 major classes, including acylcarnitines (AC), cardiolipins (CL), ceramides (Cer), cholesterol esters (CE), diacylglycerols (DAG), dihydroceramides (dhCer), free fatty acids (FFA), glucosylceramides (GlcCer), lactosylceramides (LacCer), lysophosphatidylcholines (LPC), lysophosphatidylglycerols (LPG), lysophosphatidylethanolamines (LPE), phosphatidylcholines (PC), phosphatidylethanolamines (PE), alkylphosphatidylethanolamine (PE(O)), phosphatidylglycerols (PG), phosphatidylinositols (PI), phosphatidylserines (PS), sphingomyelins (SM), triacylglycerols (TAG), and trihexosylceramides (THC) (Fig. 1a). Venn diagram analysis shows 130 species (22% of HFpEF-altered lipids) overlap between clinical phenotypes, with 469 uniquely altered in HFpEF and 102 uniquely in HFrEF (Fig. 1b). Lipid class-level enumeration (Fig. 1c) shows HFpEF exhibited substantially increased lipid species across nearly every class vs. non-failing controls but had reduced medium- and long-chain acylcarnitine species. Lipid changes in HFrEF were far less and more balanced (e.g. up and down), and had similar declines in acylcarnitines but also in total PEs and CLs compared to NF. The latter two classes were not significantly changed in HFpEF myocardium.

Unsupervised hierarchical clustering yielded 4 clusters, one predominantly HFpEF, one predominantly NF, and the other a mixture of the three groups (Fig 1d). One mixed cluster had increased TAGs, and another reduced levels. Volcano plots of differential lipid abundance between HFpEF vs. NF showed marked asymmetry with a 60:1 bias favoring accumulation over depletion (Fig. 1e). This contrasted to that for HFrEF vs. NF that had a near-balanced ratio with 130 elevated vs. 102 depleted species (1.3:1) (Fig. 1f). Direct comparison of HFpEF vs. HFrEF showed an abundance of lipids in the HFpEF myocardium (Ext. Fig. 1a).

Differentially abundant lipids in HFpEF and HFrEF were generally uncorrelated with BMI (Ext. Fig. 1b-c, unadjusted p-values show, if adjusted for multiple comparisons, Spearman with Benjamini-Hochberg, none were significant). Thus, while obesity might be expected to relate to the patterns, the data suggest the HFpEF syndrome is more central to the observed lipid abundance. In HFpEF where plasma lipids (e.g. LDL, HDL, total TAGs, and total cholesterol) were also measured (Ext. Table 2), none were correlated after FDR correction with altered myocardial lipids.

### HFpEF Exhibits Broad Lipid Accumulation with a Distinct Sphingolipid Signature

**Fig. 2.**
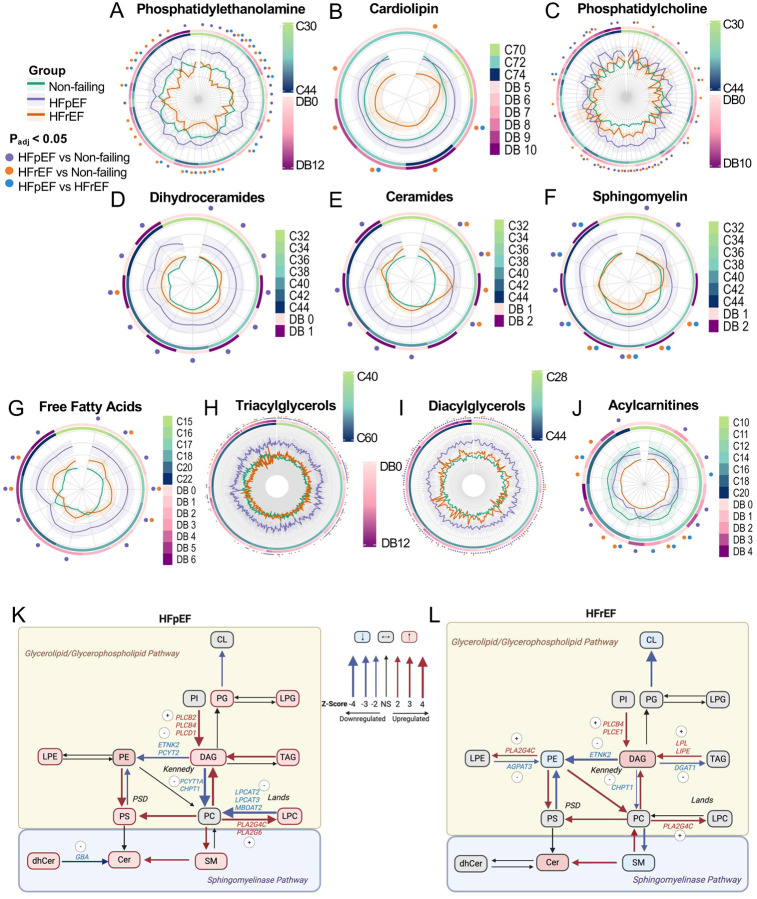
Phenotype-specific myocardial lipid remodeling in HFpEF and HFrEF. (a–j) Radial (flower) plots of lipid classes depicting z-score-normalized abundance of individual lipid species by acyl chain length (C) and double bond (DB) status for NF control (green), HFpEF (purple), and HFrEF (red). Shaded areas represent SEM. Filled circles outside the radial boundary indicate statistically significant differences with adjusted p-values of <0.05 between groups: purple = HFpEF vs. NF; red = HFrEF vs. NF; blue = HFpEF vs. HFrEF. Panels: (a) phosphatidylethanolamines (PE), (b) cardiolipins (CL), (c) phosphatidylcholines (PC), (d) dihydroceramides (dhCer), (e) ceramides (Cer), (f) sphingomyelins (SM), (g) free fatty acids (FFA), (h) triacylglycerols (TAG), (i) diacylglycerols (DAG), and (j) acylcarnitines (ACs). (k) Lipid metabolic pathway activity network for HFpEF vs. NF myocardium generated using LipidSig 2.0. Nodes represent lipid classes; directed arrows represent biochemical conversions. Arrow color and pathway z-scores indicate relative activation (red) or suppression (blue) with arrow heaviness representing z-score value. Concordant transcriptomic changes in lipid metabolism enzymes from a previously published RNA-sequencing dataset^23^ are annotated at relevant pathway nodes (red = upregulated gene; blue = downregulated gene). (l) Corresponding lipid pathway activity network for the HFrEF vs. NF myocardium.

Phosphatidylethanolamines (PEs) and cardiolipins (CLs) concentrate in the inner mitochondrial membrane and are required to maintain the high negative curvature of cristae needed for electron transport complex organization and mitochondrial membrane potential. Together they constitute the dominant non-bilayer lipid environment in which respiratory super-complexes are embedded and stabilized^16^. PEs were broadly reduced in HFrEF but increased in HFpEF (Fig. 2a), the lowest in HFrEF being PE(18:2_22:5). This is a long-chain polyunsaturated PE species structurally analogous to high unsaturated, linoleoyl-enriched CL species in the IMM. CLs were generally also reduced in HFrEF but unchanged in HFpEF (Fig. 2b). The most abundant and functionally important CL species, CL(18:2)_4_, was significantly reduced only in HFrEF (log_2_FC=−1.44)^17^. Phosphatidylcholines (PCs) are the predominant phospholipids of eukaryotic membranes and contribute to membrane structure, lipid transport, and cellular signaling; importantly, the degree of fatty acyl saturation within PC species influences membrane biophysical properties, with more saturated PCs promoting tighter lipid packing and lower membrane fluidity^18^. PCs with fewer double bonds (e.g. more saturated) tended to be increased in HFpEF more so than HFrEF myocardium (Fig. 2c).

Sphingolipids are bioactive lipids derived from condensation of serine and palmitoyl-CoA. The central hub of sphingolipid metabolism, ceramides, are established mediators of mitochondrial dysfunction, insulin resistance, and cardiomyocyte apoptosis^19^. Dihydroceramides are immediate ceramide precursors in their synthesis pathway, and sphingomyelins represent a major ceramide reservoir in membranes. We found sphingolipids, including sphingomyelins, dihydroceramides, and ceramides broadly increased in HFpEF but mostly unchanged in HFrEF, both vs. NF (Fig. 2d–f).

Triacylglycerols (TAGs) are the principal neutral lipid storage species in cardiomyocytes, while diacylglycerols (DAGs) serve as intermediates in glycerol-for phospholipid synthesis and for intracellular signaling^20^. Free fatty acids (FFAs) are the predominant substrate for mitochondrial fatty acid β-oxidation that normally accounts for 60–70% of cardiac ATP production^7^. Acylcarnitines (ACs) are obligate intermediates generated by carnitine palmitoyltransferase 1 (CPT1)-mediated esterification of fatty acyl-CoA with carnitine, enabling transport of long-chain fatty acids across the inner mitochondrial membrane for β-oxidation^21^. FFAs, TAGs, and DAGs, were broadly increased in HFpEF myocardium (Fig. 2g–i). By contrast, ACs were reduced in both HFpEF and HFrEF, more so with the latter (Fig. 2j). The rise in FFAs, TAGs, and DAGs yet reduced ACs with HFpEF contrasts to HFrEF where AC decline occurred with little upstream substrate accumulation. This suggests different sites of metabolic impairment between the HF forms, with possibly greater cellular uptake but reduced mitochondrial utilization in HFpEF, and primarily reduction of the latter in HFrEF.

To contextualize differential lipid abundance within a systems-level framework, we applied the Pathway Activity Network in LipidSig 2.0^22^, which maps untargeted lipidomics data onto known lipid biosynthetic and metabolic reaction networks to infer inter-class conversion pathway activity. The analysis uses a Z-score statistic derived from differential lipid species abundance across connected nodes to estimate directionality and relative pathway engagement between conditions. Significant changes in gene expression of metabolite conversion enzymes obtained from prior HFpEF and HFrEF myocardial transcriptomics^23^ are noted along with corresponding weighted pathway arrows. The analysis (Fig. 2k, 2l) found both HF forms favored TAG to DAG conversion, less DAG to PC (Kennedy pathway), and more PC to LPC (Lands cycle); all more favored in HFpEF. PC to SM conversion was favored in HFpEF, consistent with elevation of sphingomyelins, ceramides, and dihydroceramides in HFpEF vs. HFrEF.

### Plasma lipidomics detects fewer changes and is similar between HF forms

**Fig. 3.**
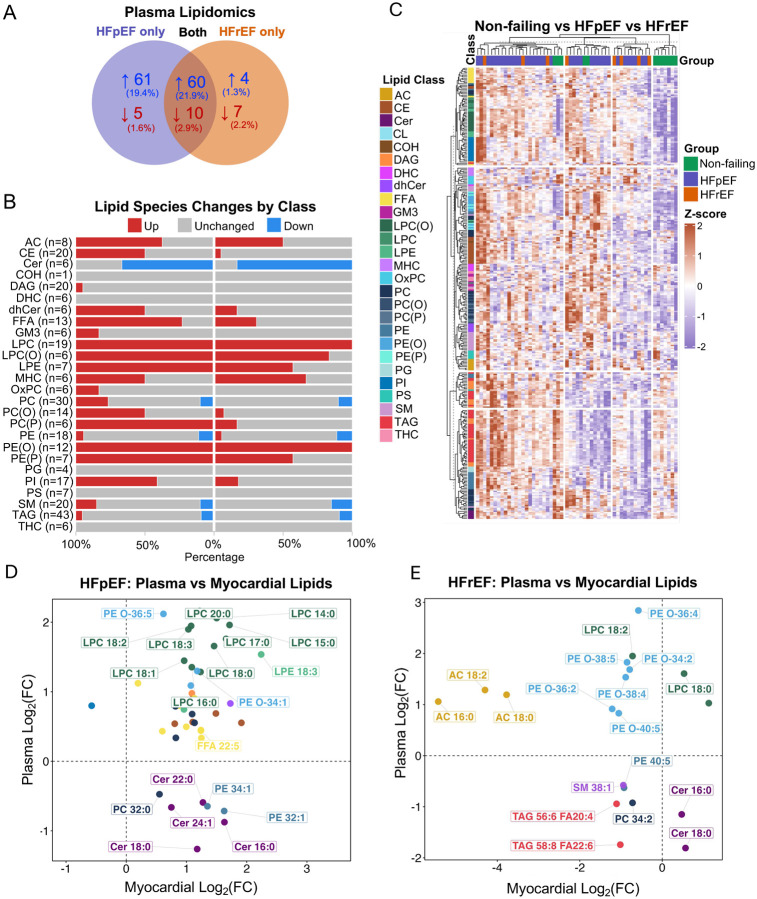
Plasma lipidomics profile in HFpEF and HFrEF. (a) Venn diagram of differentially abundant plasma lipid species in HFpEF only, HFrEF only, or concordantly changed in both. (b) Percentage of species significantly changed within lipid class in HFpEF and HFrEF vs. NF in plasma. Blue = % decreased, grey = % unchanged, red = % increased. (c) Unsupervised hierarchical clustering heatmap of plasma lipidome of log_2_-transformed, z-score-normalized lipid abundances. Sample annotations: NF (green), HFpEF (purple), HFrEF (red). Lipid class is color coded on the left of the heatmap. (d) Scatter plot correlating log_2_FC in myocardium vs. log_2_FC in plasma in HFpEF compared to NF. Only individual lipid species that were significantly changed in both myocardium and plasma (q<0.05) are shown (e). Corresponding scatter plot for HFrEF vs. NF, similarly only showing lipids meeting statistical threshold (q<0.05) in both plasma and myocardium. Lipid species abbreviations: acylcarnitines (AC), cholesterol esters (CE), ceramides (Cer), free cholesterol (COH), diacylglycerols (DAG), dihexosylceramides (DHC), dihydroceramides (dhCer), free fatty acids (FFA), GM3 ganglioside (GM3), lysoalkylphosphatidylcholine (LPC(O)), lysophosphatidylcholines (LPC), lysophosphatidylethanolamines (LPE), monohexosylceramides (MHC), oxidized phosphatidylcholines (OxPc), phosphatidylcholines (PC), Alkylphosphatidylcholine PC(O)), Phosphatidylcholine plasmalogen (PC(P)), phosphatidylethanolamines (PE), Alkylphosphatidylethanolamine (PE(O)), Phosphatidylethanolamine plasmalogen PE(P)), phosphatidylglycerols (PG), phosphatidylinositols (PI), phosphatidylserines (PS), sphingomyelins (SM), triacylglycerols (TAG), trihexosylceramides (THC).

Lipid contents of plasma from a subset of the same three human cohorts were also assayed, with a narrower lipidomic profile containing 314 species across 27 classes (Ext. Fig 1d). Clinical demographics and features of these subgroups are provided in Ext. Table 2. Of the lipid species quantified, 204 overlapped with the myocardial library and were used for comparisons. The Venn diagram shows most altered lipid species were similarly changed in HFpEF and HFrEF (Fig. 3a). Quantitative class-level analysis revealed similar changes in both HFpEF and HFrEF plasma, with elevated PE(O) and lysophosphatidylcholines (LPC) and reduced ceramides relative to NF. (Fig. 3b). Unsupervised hierarchical clustering showed overlap between HFpEF and HFrEF, whereas NF fell largely in a single cluster (Fig. 3c). Volcano plots of differential lipid abundance in plasma between HFpEF vs. NF and HFrEF vs. NF showed similar asymmetry favoring accumulation over depletion (Ext. Fig. 1e and 1f). Direct comparison of plasma lipid abundance in HFpEF vs. HFrEF revealed remarkably similar lipid profiles (Ext. Fig. 1g). This contrasted with an asymmetric increase in myocardial lipid abundance comparing HFpEF to HFrEF (Ext. Fig. 1a).

There was no significant global correlation between myocardial and plasma lipid species changes in HFpEF vs. NF (Fig. 3d) or HFrEF vs. NF (Fig. 3e). On an individual lipid species basis, however, several LPCs, PE(O), FFAs, and CEs had concordant increases in plasma and myocardium in HFpEF (Fig. 3d). They included mostly saturated LPCs (e.g., 14:0, 15:0, 18:0), and PE(O)s and FFAs of ranging double bond status. The divergence of reduced long chain acylcarnitines (LCACs) and PE(O)s in myocardium but elevation in plasma was present in HFrEF (Fig 3e).

### Non-failing heart from obese humans have few lipid or ultrastructural changes

**Fig. 4.**
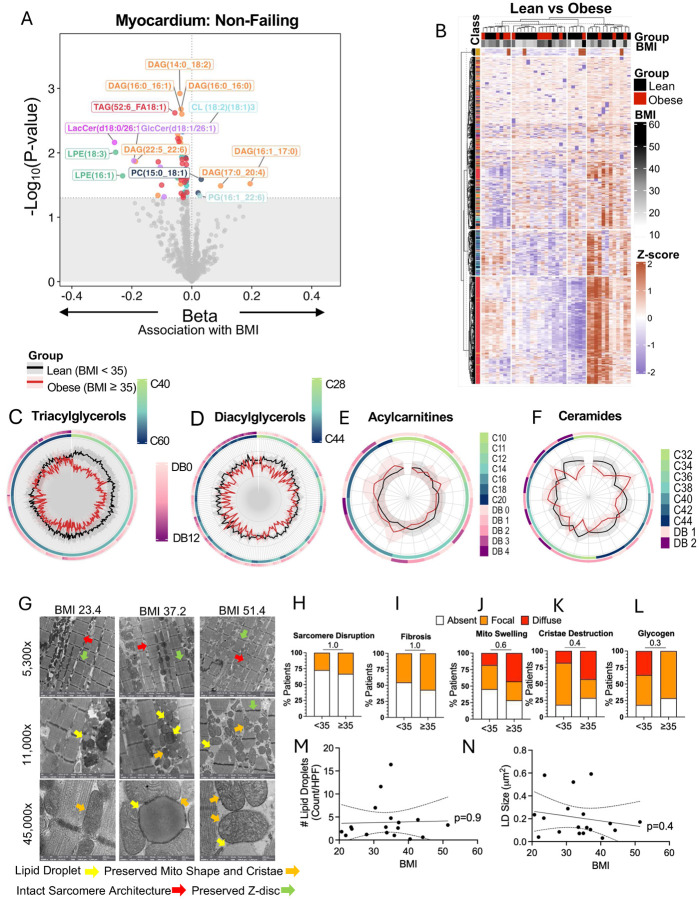
Severely obese non-failing myocardium resists lipid accumulation and maintains ultrastructural integrity. (a) Volcano plot of BMI as a continuous variable correlated with lipid species abundance in non-failing myocardium. Species above the horizontal line have slopes significantly different from zero (p<0.05). The x-axis represents the regression coefficient (β) for BMI, where species to the right of the vertical line indicate increasing lipid abundance with higher BMI and species to the left indicate decreasing abundance with higher BMI. (b) Unsupervised hierarchical clustering of non-failing myocardial lipidome; samples are annotated by BMI on a grayscale spectrum; lean (BMI <35 kg/m^2^) is black and obese (BMI ≥35 kg/m^2^) is red. Radial plots of (c) triacylglycerols, (d) diacylglycerols, (e) acylcarnitines, and (f) ceramides. Shading represents SEM. Acyl carbon chain length (C) (green to blue scale), and double bond (DB) count (pink scale) are depicted by the coloring of the outer rings of the plots. (g) Representative transmission electron micrographs of non-failing myocardium from lean (BMI 23.4 kg/m^2^), obese (BMI 37.2 kg/m^2^), and severely obese (BMI 51.4 kg/m^2^) donors at 5,300×, 11,000×, and 45,000× magnification. Scale bars as indicated. Yellow arrow = lipid droplet; orange arrow = preserved mitochondrial shape and cristae; red arrow = intact sarcomere architecture; green arrow = preserved sarcomere Z-disc. (h) Scoring of sarcomere disruption from non-failing subjects with BMI <35 kg/m^2^ (n=11) vs. BMI ≥35 kg/m^2^ (n=7). (i) Qualitative fibrosis abundance score. (j) Degree of mitochondrial swelling. (k) Cristae destruction score. (l) Glycogen deposition score. (m) Lipid droplet count per microscopic field vs. BMI. (n) Mean lipid droplet area per patient vs. BMI. Correlation statistics with BMI were calculated using simple linear regression.

Obesity is substantial in many HFpEF patients, and in animals, such obesity results in myocardial lipid accumulation. To examine if lipidomic disparities in HFpEF could result from obesity itself, NF groups were sorted by BMI (<35 kg/m^2^; median 25.2 vs. ≥35 kg/m^2^, median 39.7, Ext. Table 3) and lipidomics compared. Fig. 4a shows a volcano plot for the linear regression coefficient (beta) between individual lipid species abundance and BMI as a continuous variable. Fifty-six lipids reached significance (un-adjusted p<0.05), the majority negatively correlated with BMI, only 5 being positively correlated. None were significantly altered by BMI categorization after FDR correction. Non-hierarchical unsupervised clustering (Fig. 4b) identified 4 groups, but none aggregated lean vs. obese patients. Lipid pathway activity network analysis identified positive directionality from DAGs and LPCs to PCs (Ext. Fig 2). Radial plots show TAGs, DAGs, acylcarnitines, and ceramides were similar between lean and obese NF hearts across the range of FA chain lengths (Fig. 4c–f).

The general lack of lipid increase in NF myocardium from very obese individuals stood in marked contrast to findings in obese HFpEF. Prior ultrastructural analysis of obese HFpEF had found an abundance of glycogen and lipid droplets with positive BMI correlation, mitochondrial swelling with disrupted cristae, and sarcomere disassembly^24^. In NF myocardium, however, we found minimal lipid droplet or glycogen, and mitochondria and sarcomere structures that were preserved regardless of BMI (Fig. 4g–n). This indicates that changes observed with obese HFpEF were not related to the severity of obesity itself.

### Animal models of HFpEF or HFrEF do not phenocopy human HF lipidomics

**Fig. 5.**
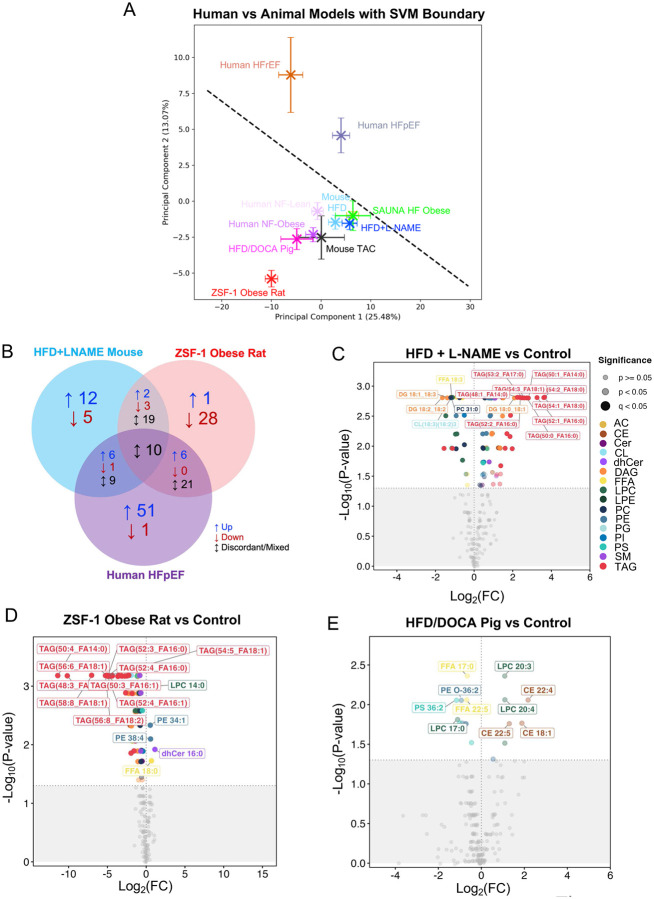
Human myocardial lipidomes are not recapitulated by animal models of HFpEF or HFrEF. (a) PCA plot using group centroids with SVM decision boundaries comparing myocardial lipidomes of human HFpEF, human HFrEF, human NF Lean, human NF obese, HFD+L-NAME mouse, ZSF-1 obese rat, SAUNA obese HFpEF model, mouse HFD obese, mouse TAC, and Göttingen minipig (HFHC diet + DOCA). Error bars signify SEM. (b) Venn diagram depicting the number of concordant significantly altered myocardial lipid species changes compared to respective species control in human HFpEF, HFD+L-NAME mouse, and ZSF-1 obese rat. (c) Volcano plot for HFD+L-NAME mouse vs. control myocardial lipidome. Smaller colored circles denote nominal significance (p <0.05), while larger colored circles denote false discovery rate (FDR)-adjusted significance (q <0.05). Faded grey circles represent lipids with nominal p >0.05. (d) Volcano plot for ZSF-1 obese rat vs. lean control myocardial lipidome. (e) Volcano plot for Göttingen minipig HFHC diet + DOCA vs. control myocardial lipidome. Lipid species abbreviations: acylcarnitines (AC), cholesterol esters (CE), ceramides (Cer), cardiolipins (CL), dihydroceramides (dhCer), diacylglycerols (DAG), free fatty acids (FFA), lysophosphatidylcholines (LPC), lysophosphatidylethanolamines (LPE), phosphatidylcholines (PC), phosphatidylethanolamines (PE), phosphatidylglycerols (PG), phosphatidylinositols (PI), phosphatidylserines (PS), sphingomyelins (SM), triacylglycerols (TAG).

The majority of biological studies of HFpEF are conducted in animal models, the most widely used model currently being mice exposed to the NOS inhibitor L-NAME and fed a high fat diet (HFD+L-NAME). Two other models, the ZSF-1 obese rat, and a Göttingen minipig fed a Western diet and treated with corticosteroids (HFHC diet + DOCA) provided alternative models and species. To test the extent to which these models capture the lipidomic profiles found in human HFpEF, myocardial tissues were run using the same assay, human NF lean and NF obese each normalized to pooled NF as used for HFpEF and HFrEF comparisons, and animal model data each normalized to their respective within-species controls. PCA with support vector machine (SVM) classification was subsequently applied to PCA coordinates using human NF and HFpEF samples to define a directional separation boundary to assess lipid remodeling patterns across models. The result (Fig. 5a) shows human HFpEF and HFrEF lipidomes occupy a distinct multidimensional space from all animal models. The two mouse HFpEF models (HFD+L-NAME and SAUNA HF Obese) clustered with obesity (HFD) only and were close to NF lean-human, whereas the pig HFpEF model lay closer to pressure-load alone (TAC) and the obese-NF human. The rat model was distinct but also furthest from human HFpEF or HFrEF. The top features driving PC1 were TAGs and DAGs, that showed the greatest overlap between HFpEF and animal models of obesity with or without HFpEF. Top features driving PC2, that largely separated human HFpEF and HFrEF from the animal models, were phospholipids, FFAs, and CLs (Ext. Fig. 3a-b).

Venn diagram analysis of differentially altered lipid species between human HFpEF, HFD+L-NAME mouse, and ZSF-1 obese rat revealed minimal concordance, with few species altered in the same direction in both human and animal datasets (Fig. 5b). Volcano plots for individual animal HFpEF models confirmed that HFD+L-NAME mice have TAG accumulation but without the broad sphingolipid, phospholipid, acylcarnitine, and free fatty acid dysregulation found in human HFpEF (Fig. 5c). The ZSF-1 obese rat had a narrow lipid remodeling signature with depletion of TAGs (Fig. 5d). The Göttingen minipig model did not capture ceramide and acylcarnitine signatures found in human HFpEF (Fig. 5e). Other control models, including trans-aortic constriction pressure overload, a widely used model of HFrEF, showed essentially no significant myocardial lipidomic changes unlike human HFrEF (Ext. Fig. 4a). High-fat diet induced obesity in mice resulted in TAG accumulation (Ext. Fig 4b) that had been found minimal in NF-hearts from very obese humans. Thus overall, animal models of HFpEF, HFrEF, or obesity, did not capture myocardial lipidomic signatures found in their human counterparts.

## DISCUSSION

This study provides the first comprehensive analysis of the human myocardial lipidome in HFpEF, contrasting findings to late-stage HFrEF and NF hearts including those from very obese individuals. By combining this broad human tissue resource with a high-throughput LC-MS/MS platform capable of quantifying >1,000 unique molecular species from limited biopsy material, we provide greater biochemical resolution than prior human myocardial studies that focused on selected lipid classes such as acylcarnitines, ceramides, or triglycerides^13,14^. This breadth is important because myocardial lipid remodeling is unlikely to be captured adequately by isolated lipid subclasses, but requires lipidome-scale analysis to reveal coordinated biochemical changes. To our knowledge, only one prior study has reported even limited human ventricular lipidomics from HFpEF but included only 4 non-failing controls with uncertain cardiac function, had a far smaller lipid panel, and no comparisons to HFrEF^25^. Comprehensive myocardial lipidomics has also not been done in established HFpEF animal models, with data limited to selective lipid droplet quantification, targeted phospholipid analysis, and cardiolipin profiling^26,27^. The present dataset therefore fills a critical void in both human and preclinical HFpEF lipidomic literature. There are several major findings. First, HFpEF is defined by broad multi-class lipid accumulation with a signature that is quantitatively and qualitatively distinct from HFrEF. Second, HFpEF-specific lipid dysregulation is largely independent of body mass index and uncorrelated with conventional clinical parameters. Third, severely obese non-failing hearts do not accumulate myocardial lipids as in obese-animal studies, challenging the notion that this morbidity itself induces lipotoxicity as a driver for obesity-associated HFpEF.

The ~60:1 ratio of lipid accumulation vs. depletion in HFpEF myocardium spanning phospholipids, sphingolipids, ceramides, neutral lipids, and free fatty acids, establishes a severe and distinctive metabolic phenotype. That these changes were broadly uncorrelated with the severity of obesity reflected by BMI or with plasma lipidemia highlights the role of other key and as yet unidentified features of the HFpEF heart and syndrome. A defining feature of this lipidome was broad coordinated elevation of all three sphingolipid subclasses that are not observed with HFrEF. Ceramides are structurally defined by a sphingosine backbone N-acylated with a fatty acid chain of varying length (typically C14–C26). Ceramide accumulation in the failing myocardium has been shown to promote cardiomyocyte apoptosis via activation of protein phosphatase 2A (PP2A) and protein phosphatase 1 (PP1), leading to dephosphorylation and inactivation of Akt, and to direct activation of the mitochondrial apoptotic pathway through Bax translocation^14,28^. The biological potency of ceramides is highly chain-length dependent. C16:0 ceramide (generated primarily by CERS5 and CERS6) being particularly implicated in insulin resistance and pro-apoptotic signaling^29^. The elevation of this and other ceramide species supports the concept of lipotoxicity particularly in HFpEF myocardium.

The coexistence of elevated fatty acid substrates (FFAs, TAGs, DAGs) but reduced acylcarnitines in HFpEF myocardium supports metabolic uncoupling between FA uptake and metabolism that is not found in HFrEF. This pattern in HFpEF is consistent with increased lipid droplets found in EM images, and depression of various components of mitochondrial FA transport and catabolism^24^. This metabolic bottleneck could in part explain the energetic insufficiency in HFpEF myocardium^30^, indicating fatty acids are not effectively entering mitochondria for oxidation, and as a result TAGs, DAGs, and ceramides accumulate generating a lipotoxic milieu. The shared acylcarnitine depletion in HFpEF and HFrEF suggests impaired fatty acid oxidation as a common heart failure endpoint with HF, but the divergent upstream substrate profiles indicate distinct pathogenic mechanisms. Isotope tracing and flux studies in experimental HFrEF models (e.g. TAC, pressure-overload) have consistently demonstrated global suppression of fatty acid oxidation with a metabolic shift toward glucose utilization^6^. While flux studies have not been performed in human HFpEF myocardium, the patterns we observe suggest a fundamentally different metabolic architecture: in HFpEF, fatty acids seem to be delivered but cannot be efficiently catabolized. Moreover, prior human myocardial metabolomics analyses suggest that the HFpEF heart does not efficiently shift to use of alternative fuels such as the substrates from glycolysis, the tricarboxylic acid cycle, and branched-chain amino acids^12,25^. So, despite likely impaired fatty acid oxidation and lipid excess, the HFpEF heart may not have effective ways to dispose of these toxic intermediates.

The lack of lipid accumulation and associated ultrastructural defects in NF hearts of severely obese humans is an important finding by itself. It stands in contrast to the rodent literature, where high-fat diet feeding in mice or rats consistently produces substantial lipid droplet accumulation, intramyocellular triglyceride accumulation, phospholipid shifts, and lipid-associated ultrastructural changes^31,32^. Large animal models of obesity are far less studied, but pig obesity models report myocardial TAG accumulation^33^. Importantly, murine hearts rely heavily on glucose oxidation^34^ and have higher heart rates, resulting in different steady-state lipid turnover kinetics. Isoform differences in key lipid handling proteins, such as fatty acid translocase (CD36), adipose triglyceride lipase (ATGL) and lipid droplet associated proteins may confer different lipid sequestration and mobilization capacities between species. It is possible that pathway adaptive responses may also be more robust in humans than in rodents, buffering against DAG accumulation and downstream lipotoxic signaling. Although not a substitute for lipid flux measurements, the pathway activity network analysis performed in this study does suggest modifications in the NF hearts from obese individuals, with a shift from DAGs and LPCs toward PCs. This is opposite to the direction in HFpEF and HFrEF. Thus, while obesity may provide a metabolic substrate, other insults likely result in loss of an adaptive catabolic capacity to instead favor lipid accumulation. Indeed, only a minority of the ~10–20% of adults in the U.S. with severe obesity develop HFpEF.

The lack of established animal HFpEF models to capture the myocardial lipid remodeling signature of human HFpEF and HFrEF has translational importance. Animal HFpEF models report myocardial lipid droplet accumulation and selected phospholipid changes^35,36^ but do not capture human lipidome complexity. This may relate to species differences in cardiac lipid metabolism, adaptations to hyperlipidemic stress, or less severe HF component of these models. While the models capture EF preservation, diastolic dysfunction, and obesity (often less than in humans), the conditions differ from human HFpEF, and caution is advised when altering their lipid pathways and regulation.

Our study has its own limitations. It was cross-sectional, precluding determination of whether lipid remodeling precedes, accompanies, or follows HFpEF development. The non-failing controls are from organ donors, and while we excluded individuals with known cardiac disease, acute brain death may transiently alter systemic, and specifically cardiac, metabolism. Our HFpEF cohort was predominantly obese (median BMI 37.3 kg/m^2^), reflecting contemporary HFpEF epidemiology but limiting generalizability to lean HFpEF, which may have a distinct lipid remodeling signature. Lipid measurements represent steady-state abundances rather than metabolic flux which requires use of isotope tracing. Such studies would more conclusively establish whether acylcarnitine depletion reflects reduced β-oxidation flux or altered steady-state turnover but have yet to be performed and will be limited in scope even with fresh but still biopsy-sized myocardium. In vivo infusion of stably labeled lipids with carbon tracing in subsequent biopsies is feasible, though not previously reported for any human condition, and costly. Our HFpEF cohort was predominantly Black/African American (55%) and will require proof of generalizability to other populations with future studies. Finally, the HFrEF cohort (n=13), while sufficient for discovery-level analyses, limited subgroup analyses for that phenotype.

In conclusion, we establish broad lipid dysregulation and accumulation in the human HFpEF heart that is distinct from more balanced selective remodeling in HFrEF. This specific lipid dyshomeostasis is neither induced by obesity nor reflected in plasma lipidomics. It is also not recapitulated by accepted pre-clinical HFpEF models revealing a translational gap that remains to be remedied. Our findings reframe HFpEF as a disease of failed lipid metabolic adaptation, not only lipid excess, and challenge the paradigm that obesity itself is centrally driving lipotoxicity that in turn leads to the HFpEF heart.

## METHODS

### Study Population, Biopsy Acquisition, and Processing

Patients referred to the Johns Hopkins HFpEF Clinic for evaluation were enrolled if they met clinical consensus criteria^37^ for the diagnosis of HFpEF and had undergone hemodynamic assessment by right heart catheterization with endomyocardial biopsy. The primary diagnostic criteria were clinical signs and symptoms of heart failure, an ejection fraction (EF) ≥50%, and hemodynamic evidence of elevated left-sided filling pressures (pulmonary capillary wedge pressure ≥15 mmHg at rest or ≥25 mmHg with exertion). Plasma and endomyocardial biopsy samples were collected from HFpEF subjects between August 2017 and November 2023. Blood collection and endomyocardial biopsies were performed under a research protocol approved by the Johns Hopkins Institutional Review Board, and informed consent was provided by all patients. In the myocardial lipidomic analysis, n=40 non-failing controls, n=29 HFpEF, and n=13 HFrEF subject samples were assessed (Ext. Table 1). Plasma lipidomics were analyzed in n=12 NF controls, n=37 HFpEF, and n=8 HFrEF subjects (Ext. Table 2). HFpEF myocardial tissue was obtained by mid-septal endomyocardial biopsies (~2–3 mg) from the right ventricular side of the interventricular septum as previously described^23^. Samples were rapidly placed in liquid nitrogen. Non-failing control hearts were obtained from brain-dead organ donors whose hearts had no identifiable disease but were not used for transplantation, most often due to advanced age, as previously described^38^. Explanted HFrEF hearts from patients undergoing cardiac transplantation were also studied. For these whole-heart NF and HFrEF explants, hearts were retroperfused with cold cardioplegic solution in vivo, extracted, and immediately placed in cold calcium-free buffer; myocardial samples (~1 g) from the LV free wall were placed into liquid nitrogen. Estimated glomerular filtration rate was calculated using the CKD-EPI equation^39^.

### Mass Spectrometry-Based Lipidomic Assessment

#### Chemicals

Tetrahydrofuran, methanol, ultrapure water, butanol, chloroform, Tyrode’s salt solution, and ammonium formate were purchased from Thermo Fisher Scientific. All solvents were of HPLC-MS grade. UltimateSPLASH ONE and standards PC 9:0/9:0, C3-acylcarnitine (d5), and C14-acylcarnitine (d3) were purchased from Avanti Polar Lipids. Standards MHC 16:0 (d3) and DHC 16:0 (d3) were purchased from Cayman Chemical.

#### Tissue Homogenization

Tissue was homogenized in reinforced 2 mL screw-cap tubes containing 2.8 mm ceramic beads with Tyrode’s salt solution (pH 7.6) at a concentration of 175 mg/mL using an Omni Bead Ruptor 24 (Omni International Inc., USA). The homogenate was diluted to 25 mg/mL and stored at −80°C^40,41^.

#### Lipid Extraction

Lipid extraction was performed from tissue and plasma using a modified Folch method^42,43^. Briefly, a 10 μL aliquot of each sample was added to 1 mL of chloroform/methanol (2:1, v/v), followed by 90 μL of PBS and 10 μL of a 1:10 dilution of UltimateSPLASH ONE (Avanti Polar Lipids), including additional internal standards at a final concentration of 0.3 nmol/mL. Samples were vortexed and centrifuged to induce phase separation, after which the organic layer was collected and dried under a gentle stream of nitrogen. The resulting lipid extract was reconstituted in 50:50 (v/v) butanol/methanol and stored at −80°C until analysis.

#### HPLC

All analyses were performed using reverse-phase liquid chromatography with the following solvent system: solvent A (tetrahydrofuran/methanol/water, 20:20:60, v/v/v) and solvent B (tetrahydrofuran/methanol/water, 75:20:5, v/v/v), each containing 10 mM ammonium formate. A 1 μL aliquot of each sample was injected onto a Zorbax RRHD Eclipse Plus C18 column (2.1 × 50 mm, 1.8 μm; Agilent) using a UFLC system (Shimadzu) at a flow rate of 300 μL/min with the column oven maintained at 50°C. The gradient began at 100% solvent A, with solvent B increased linearly from 0% to 100% over 8 min, held at 100% for 2.5 min, and returned to 0% over 30 s (total run time 11.1 min). For free fatty acid analysis, solvent B was increased from 50% to 100% over 2.5 min, held for 1.5 min, and returned to 50% over 1 min.

#### Mass Spectrometry

The HPLC system was coupled to a Sciex 7500+ triple quadrupole mass spectrometer operated in scheduled multiple reaction monitoring (sMRM) mode, targeting 1,100 analytes in positive ion mode and 1,400 analytes in negative ion mode. Source parameters: curtain gas, 45 psi; ion source gas 1, 45 psi; ion source gas 2, 70 psi; collision gas, medium; source temperature, 400°C; ion spray voltage, +5,500 V (positive) and −4,500 V (negative). Data acquisition and processing were performed using SciexOS software (version 3.4.5), with quantification based on comparison of analyte peak areas to those of corresponding internal standards.

For cardiolipin and free fatty acid analyses, the HPLC system was coupled to a 4000 QTRAP triple quadrupole mass spectrometer with a Turbo V electrospray ionization source (AB Sciex) operated in sMRM in negative ion mode. Source parameters: curtain gas, 26 psi; ion source gas 1, 40 psi; ion source gas 2, 30 psi; collision gas, medium; source temperature, 500°C; ion spray voltage, −4,500 V. Data were acquired using Analyst software (version 1.6; AB Sciex) and quantified using MultiQuant software (version 2.1; AB Sciex) by comparing analyte peak areas to the internal standard PC(9:0/9:0).

### Statistical Analysis

Lipid species abundances were normalized to tissue weight prior to downstream analyses. Tissue weight-normalized lipid abundance values were subsequently compared between groups using two-sided Mann–Whitney U tests. P values were adjusted for multiple comparisons using the Benjamini–Hochberg false discovery rate (FDR) method, with an FDR threshold of 5% considered statistically significant. Continuous clinical variables are presented as median (interquartile range) and compared using the Kruskal–Wallis test with Dunn’s post-hoc test (three-group comparisons) or Mann–Whitney U test (two-group comparisons). Categorical variables are presented as n (%) and compared using Fisher’s exact test or χ^2^ test. Statistical analyses and simple linear regression analyses were performed using GraphPad Prism.

### Heatmaps

Prior to heatmap generation, multivariate outlier detection was performed using Mahalanobis distance calculated from the first five principal components derived from principal component analysis (PCA), which captured the major sources of variation within the dataset. Samples exceeding the significance threshold (α = 0.05) were classified as outliers and excluded. A total of 8 outliers were identified among 82 samples, including 4 HFpEF samples (4/29), 0 HFrEF samples (0/13), and 4 non-failing controls (4/40). Heatmaps were generated using log_2_-transformed normalized lipid abundance data with row-wise z-score normalization. Unsupervised hierarchical clustering was performed on both lipid species and samples using the ComplexHeatmap package in R.

### Z-Score Radial Plots

Z-scores were calculated from normalized lipid abundance values by centering each lipid species to the population mean and scaling to the population standard deviation across all samples and disease groups. For flower plot visualization, lipid species were grouped by lipid class, and group-wise mean ± SEM z-scores from non-failing, HFpEF, and HFrEF samples were displayed using radial plots generated in R.

### Volcano Plots

For human data, volcano plots were generated by plotting log_2_ fold change (log_2_FC) against the negative log_10_-transformed adjusted p-value (−log_10_adj. p-value). Log_2_FC was calculated as the log_2_ ratio of the mean lipid abundance in the comparison group to the mean lipid abundance in the reference group. Statistical significance was determined using two-sided Mann–Whitney U tests on tissue-normalized lipid abundance values, and p-values were adjusted for multiple comparisons using the Benjamini–Hochberg false discovery rate (FDR) method. For pre-clinical data, volcano plots were generated by plotting log_2_ fold change (log_2_FC) against the negative log_10-_transformed nominal p-value (−log_10_p-value). Statistical significance was performed as previously mentioned, and nominal p-values were taken into consideration for plotting. Lipids with p<0.05 were highlighted using semi-transparent points, while lipids meeting the FDR significance threshold (q<0.05) were shown as fully opaque points to distinguish nominally significant from FDR-significant species. Volcano plots were generated in R using the ggplot2 package, and selected significant lipid species were annotated.

### Lipid Pathway Activity Analysis

Lipid metabolic pathway activity analysis was performed using LipidSig 2.0^22^. Normalized lipid abundance data were uploaded to the platform’s Differential Expression Analysis tool. The resulting .rds output file was then imported into the Pathway Network Activity module for pathway activity scoring and network visualization. Lipid classes were represented as nodes and biochemical conversions as directed edges, with edge color and pathway z-scores indicating relative pathway activation or suppression.

### Plasma Lipidomics and Myocardial Lipidomics Correlation

Raw lipid abundance values were quantified in 314 unique species in the human plasma (Ext. Fig 1d), while 1,058 unique species were detected in the human myocardium (Fig 1a). Of the plasma lipid species quantified, 204 overlapped with the myocardial library and were used for comparisons. Among the species that met statistical significance (q<0.05 HFpEF vs NF and q<0.05 HFrEF vs NF) in both plasma and myocardial pools were compared by direction and magnitude of FC.

### Transmission Electron Microscopy

Myocardial samples from 18 non-failing controls were used for TEM analysis with BMI ranging from 20.7–51.4. n=11 had BMI<35 and n=7 had BMI >35. During tissue harvest, samples were fixed in 3.0% glutaraldehyde in 0.1 M sodium phosphate buffer (pH 7.3) were post-fixed in 1% osmium tetroxide, dehydrated through graded ethanols, and embedded in Epon (PolySci) resin. Samples were polymerized overnight at 60°C. Thin sections (60–90 nm) were cut using a diamond knife on a Leica EM UCT ultramicrotome and picked up with 2×1 mm Formvar copper slot grids. Grids were stained with 3% uranyl acetate followed by lead citrate and observed with a ThermoFisher Talos L120C at 120 kV. Images were captured with a ThermoFisher Ceta camera (16 MP cMOS). For each sample, 5 random fields were imaged at 5,300×, 11,000×, and 45,000× magnification by a blinded imager. Tissue processing and imaging were performed at the Johns Hopkins University Microscope Facility. Lipid droplets were identified as circular electron-lucent structures and quantified using ImageJ by blinded observers (V.B. and K.T.). Lipid droplet area was determined by tracing individual organelles using the freehand selection tool; a mean lipid droplet area was calculated by quantifying individual lipid droplets within each field, and these per-field counts were then averaged across five fields per subject to yield a single mean area per subject. All images were reviewed by a clinical cardiac pathologist (C.I.D.) who was blinded to clinical data and subject group assignments. Sarcomere disruption, fibrosis, mitochondrial swelling, cristae destruction, and glycogen content were scored as 0=absent, 1=rarely seen, 2=frequently seen. Pairwise comparisons of categorical variables were performed using Fisher’s exact test.

### Transcriptomic Integration

Transcriptomic data from a previously published bulk RNA-sequencing dataset of HFpEF, HFrEF, and non-failing human myocardium^23^ were integrated with lipidomic pathway analysis. Differentially expressed genes encoding lipid metabolism enzymes were mapped to LipidSig 2.0 pathway nodes, and directional concordance between transcriptomic and lipidomic pathway changes was assessed qualitatively. Genes with adjusted p<0.05 were considered concordant.

### Animal Model Lipidomics

Animal models were generated in separate laboratories (HFD+L-NAME, HFD, and TAC mouse models from the D.A. Kass laboratory; ZSF-1 rat and minipig models from the D.J. Lefer laboratory; and SAUNA mouse model from the F. Sam laboratory) in accordance with their respective institutional animal care and use committee guidelines. Raw lipid abundance values were normalized to tissue weight prior to analysis. For cross-species comparisons, 202 lipid species with coverage across both human and animal datasets were included. Log_2_ fold-change values were calculated relative to the corresponding control group within each dataset. PCA was performed in Python using the scikit-learn package to evaluate global lipid remodeling patterns across datasets. Support vector machine (SVM) classification was subsequently applied to the PCA coordinates using human non-failing and HFpEF samples to define a directional separation boundary, which was then used to assess the similarity and directionality of lipid remodeling patterns across experimental models. P-values were calculated for each lipid species and q-values were subsequently generated by Benjamini–Hochberg correction to control the false discovery rate.

#### L-NAME+HFD Mouse Model

Eight-week-old C57BL/6N mice (Charles River Laboratories, Wilmington, MA, USA) were treated with L-NAME in the drinking water (0.5 g/L; Sigma-Aldrich) and a 60% high-fat diet (D12492) for 16 weeks (n=8) and compared with wild-type chow-fed controls (2916, Teklad, n=5) aged for the same duration. Mice were given unrestricted access to food and maintained on a 12-h light/dark cycle. Controls were age-matched wild-type C57BL/6N mice^44^.

#### High-Fat Diet Mouse Model

Eight-week-old C57BL/6J mice (The Jackson Laboratory, Bar Harbor, ME, USA) were treated with a 60% high-fat diet for 16 weeks (n=4) and compared with wild-type chow-fed controls (2916, Teklad, n=5) aged for the same duration. Mice were given unrestricted access to food and maintained on a 12-h light/dark cycle.

#### Transverse Aortic Constriction Mouse Model

Eight-week-old C57BL/6J mice (The Jackson Laboratory, Bar Harbor, ME, USA) were subjected to transverse aortic constriction (n=4) or sham (n=5) for 8 weeks. Mice were given unrestricted access to normal chow diet and maintained on a 12-h light/dark cycle.

#### SAUNA Mouse Model

Seven-week-old C57BL/6J male mice (The Jackson Laboratory, Bar Harbor, ME, USA) were fed an obesogenic 60% high-fat diet (D12492; Research Diets Inc., New Brunswick, NJ, USA) or control diet for 16 weeks and subsequently underwent HFpEF induction using the SAUNA model with salty drinking water (1% NaCl), unilateral nephrectomy, and chronic exposure to *d*-aldosterone (0.30 μg/h; Sigma-Aldrich, St. Louis, MO, USA, A9447) (n=5/group)^45^.

#### ZSF-1 Rat Model

Obese ZSF-1 rats (cross between female Zucker diabetic and spontaneously hypertensive male, n=5) and lean ZSF-1 lean controls (Charles River Laboratories, Wilmington, MA, USA, n=10) were purchased at 8 weeks of age and aged to 24 weeks. Rats were given unrestricted access to food (Purina 5001; Charles River Laboratories) and maintained on a 12-h light/dark cycle.

#### Göttingen Minipig HFpEF Model

Fourteen-month-old female Göttingen minipigs were fed a 50/50 (wt/wt) mixture of regular miniswine diet (8753 Teklad Miniswine Diet; Envigo, Indianapolis, IN, USA) and a custom western diet enriched in fat, fructose, cholesterol, and salt (9GCZ TestDiet, St. Louis, MO, USA) and treated with a subcutaneous desoxycorticosterone acetate (DOCA) depot (50 mg/kg, 200 mg pellets, 60-day release) for 20 weeks (n=5). Age and sex matched pigs on normal chow without DOCA treatment were used as controls (n=6). At the time of tissue collection, ejection fraction was >50%, LV end-diastolic pressure was elevated, and pulmonary capillary wedge pressure was ≥15 mmHg, meeting clinical criteria for HFpEF^46^.

## Supplementary Material

Supplementary Files

This is a list of supplementary files associated with this preprint. Click to download.

- SupplementalMaterial.pdf
